# Blood Pressure, Sleep Quality and Fatigue in Shift Working Police Officers: Effects of a Twelve Hour Roster System on Cardiovascular and Sleep Health

**DOI:** 10.3390/ijerph13020172

**Published:** 2016-01-29

**Authors:** Jaymen L. Elliott, Sara Lal

**Affiliations:** Neuroscience Research Unit, School of Life Sciences, University of Technology Sydney, Broadway, New South Wales 2007, Australia; jaymen.elliott@live.com

**Keywords:** shift work, blood pressure, sleep, fatigue, police

## Abstract

*Background:* Police officers have been reported to exhibit a high incidence of pathologies, which present prematurely in an otherwise healthy population. Shift work has also been associated with an increased risk of cardiovascular and sleep disorders, attributable to its propensity for circadian rhythm dysfunction. However, contention exists as to whether shift work has a direct effect upon blood pressure (BP) regulation. *Methods:* This cross-sectional study sought to determine changes in BP and associations with the overall sleep quality and fatigue in 206 general duties police officers (*n* = 140 males) of the New South Wales Police Force in Australia. The subjects’ BP was assessed before and after their twelve hour shift, during which time they also completed the Lifestyle Appraisal Questionnaire, Pittsburgh Sleep Quality Index (PSQI), Epworth Sleepiness Scale and Fatigue Severity Scale (FSS). *Results:* Poor sleep quality (PSQI) and fatigue severity (FSS) were found to predominate in the sample (69% and 51% respectively). Although there was no change in BP for male participants, female officers’ systolic blood pressure (SBP) was found to increase significantly across the shift (*p* < 0.001), but with no change found in females’ diastolic blood pressure (DBP). Finally, higher pre and post-shift SBP (*r* = −0.26, *p* = 0.001; *r* = −0.25, *p* = 0.001, respectively) and DBP (*r* = −0.26, *p* = 0.001; *r* = −0.26, *p* = 0.001, respectively) were significantly correlated with lower FSS scores after accounting for age, waist-hip ratio and lifestyle risk factors. *Conclusions:* Based on these preliminary findings, there was a significant increase in SBP of female police officers after shift work, while BP and fatigue levels in all police officers were strongly related. Moreover, the predominating poor sleep quality and impact of fatigue in this sample remain a concern. Further research is required to ensure the physiological welfare of police officers, while strategies must be implemented to manage the detrimental effects shift work may be having upon their cardiovascular and sleep health.

## 1. Introduction

Although policing is often romanticized, it is an inherently demanding occupation. Police officers have been shown to exhibit an increased prevalence of fatigue, which may predispose them to greater frequency of accidents and workplace injuries [1]. In Australia, the New South Wales (NSW) Police Force employs flexible rostering guidelines that result in predominating twelve hour shifts in a two day and two night cycle [2]. Concerns have been raised over “extended work hours” [3], specifically the deleterious effects upon health and sleep quality that may potentiate fatigue and sleepiness. Despite being used synonymously, due to symptomatic similarities, sleepiness and fatigue have been found to be distinct [4] and will compromise an individual’s performance. The suggested ergonomic and social benefits notwithstanding [5], shift work has been frequently associated with an increased risk of cardiovascular and metabolic pathologies [6,7,8], as well as higher reported levels of fatigue among shift working populations [9]. However there is heavy contention in the available literature as to whether shift work has a direct effect upon an individual’s blood pressure (BP) and subsequent risk for hypertension [10]. Circadian rhythm dysfunction attributable to shift work has been linked to alterations in hormone levels, worsening lifestyle risk factors and overactivation of the sympathetic nervous system [11,12], which are all known risk factors for hypertension and fatigue. Further, the relationship between sleep and cardiovascular health [13] requires additional attention, especially the interplay between fatigue and blood pressure [14]. Finally, evidence suggests police officers will experience different levels of stress and sleep quality based on their sex and position within the profession [15]. For these reasons, this study sought to determine changes in BP and the associations with overall sleep quality and fatigue of shift working police officers in NSW. Comparisons were also made between general duties police officers based on their sex, shift and occupational rank.

## 2. Methods

### 2.1. Participants

This cross-sectional study was conducted over two years and 285 Australian general duties police officers were recruited. Assessment took place at nine local area commands (LAC) of the NSW Police Force within the Sydney area, including Campsie, Eastwood, Leichhardt, Macquarie Fields, Marrickville, Newtown, Northern Beaches, North Shore and Quakers Hill. Seventy nine participants were excluded from the study based on various exclusion criteria (>10 cigarettes or standard drinks daily, ongoing medication use or a pre-existing chronic illness). The remaining 206 police officers (*n* = 140 males) were actively working twelve hour shifts. Subjects were assessed while working a day shift (*n* = 114) or night shift (*n* = 92), with turnover times roughly 06:00 and 18:00. Recruitment was on a voluntary basis, with one researcher attending LAC’s and administering the study protocol (Section 2.3). This study was approved by the institution’s Human Research Ethics Committee (HREC: 2014-000110).

### 2.2. Measures

A number of tools were used in this study to collect subjects’ demographic, cardiovascular and sleep-related information. BP was measured three times in a quiet, seated position [16] using an automated BP monitor (Livingstone, OMRON IA1b, Kyoto, Japan), with assessment time falling between 5–7 am and 5–7 pm depending on their twelve hour roster. The officers’ Waist-Hip Ratio (WHR) was measured, with abdominal obesity defined as above 0.90 for males and 0.85 for females [17]. The Lifestyle Appraisal Questionnaire Part 1 (LAQ, 22 items) [18] is a multifactorial perspective tool to assess an individual’s lifestyle risk factors, whereby a greater score indicates a higher risk of developing various pathologies. Overall sleep quality was determined using the Pittsburgh Sleep Quality Index (PSQI, 18 items) [19], which produces a global score between 0 and 21 where a score greater than 5 indicates poor sleep quality. This is based on seven components, which include subjective sleep quality, sleep onset latency, sleep duration, sleep efficiency, sleep disturbances, usage of sleep medications, and daytime dysfunction. Subjects also completed the Epworth Sleepiness Scale (ESS, eight items) [20] which assesses the likelihood of dozing in specified situations, thereby producing a score between 0 and 24 with scores ≥10 indicating chronic sleepiness. Finally, the Fatigue Severity Scale (FSS, nine items) [21] was used to determine to what degree fatigue impacts upon individual’s lives. Utilizing a Likert scale, this provides a total score from 9 to 63 with scores ≥36 indicating severe fatigue symptomology.

### 2.3. Protocol

After obtaining informed consent, participants completed an introductory questionnaire which recorded the following; age, years of active service, waist-hip ratio (WHR) and exclusion criteria information (Section 2.1). Before commencing their shift, subjects had their BP measured, before being excused to continue their regular duties, during which time they were asked to complete a small questionnaire battery (Section 2.2). After finishing their twelve hour shift, subjects were advised to return for post-shift BP assessment and asked to submit their completed questionnaires.

### 2.4. Statistical Analyses

Data were analysed using Statistica Version 8 software (StatSoft Inc., Tulsa, OK, USA) with change across shift assessed using dependent sample *t*-tests. To address the potentially confounding variables of age, WHR and lifestyle risk factors (LAQ) on BP [22,23], corrected models were employed for all subsequent analyses of the sample. This included analysis of covariance (ANCOVA) to identify differences between the sexes and partial correlations to elucidate significant associations of sleepiness and fatigue measures with BP. Further, the same analyses were employed to determine if there were differences between individuals assessed on a day or night shift. Finally, multivariate analysis of covariance (MANCOVA) was also used to investigate differences between subjects based on their occupational rank (Section 3.3), followed by a Fisher LSD Post-Hoc analysis.

## 3. Results

### 3.1. Total Sample, Sex & Shift Comparisons

Analysis was performed on data obtained from 206 police officers (Table 1). While there was no significant change in their diastolic blood pressure (DBP) across the shift, a significant increase in systolic blood pressure (SBP) (*p* < 0.05) was observed for the total sample (from 126.86 ± 12.33 mmHg to 128.11 ± 12.13 mmHg). With respect to lifestyle risk factors (LAQ Part 1) and sleepiness levels (ESS), the sample was predominantly within normal ranges. However, the mean PSQI global score of 6.83 ± 3.58 indicated a substantial amount of police officers had poor sleep quality; approximately 69% scored above the PSQI’s cut-off score of five. Further, 51% of subjects scored above the threshold score of ≥36 for severe fatigue impact, represented by the mean FSS score of 36.02 ± 10.36. When comparing police officers based on sex using ANCOVA, males (*n* = 140) were found to have significantly higher WHR (*p* < 0.001), SBP (*p* < 0.001) and DBP (*p* < 0.001) across shift than their female counterparts (*n* = 66). Further, female officers were on average significantly younger (*p* < 0.05) and had served fewer years as police officers than males (*p* < 0.05). Although there was no significant change in male officers’ BP from pre to post-shift, female officers’ SBP was found to significantly increase (from 117.52 ± 10.87 mmHg to 120.59 ± 10.99; *p* < 0.001) across the shift, with no significant change observed in DBP. However, the effect size of this comparison was relatively small (*d* = −0.28). Finally, when comparing subjects who were working a day or night shift at the time of assessment, the same ANCOVA analyses identified night officers had a significantly higher SBP (*p* < 0.03) than their day counterparts (124.82 ± 12.13 mmHg and 129.65 ± 11.88 mmHg respectively). The total questionnaire scores did not differ significantly for males and females, or between day and night shift subjects.

### 3.2. Significant Associations

Partial correlations were performed between BP measures taken pre and post-shift and questionnaire data for the total sample (Table 2). As detailed in Section 2.4, subjects’ age, WHR and lifestyle risk factors (LAQ) were designated as control variables in the partial correlation analysis. Higher pre-shift SBP was found to be solely linked with decreasing fatigue severity (FSS) (*r* = −0.26, *p* < 0.01). Similarly, higher post-shift SBP was significantly associated with lower fatigue severity (*r* = −0.25, *p* < 0.01) as well as better overall sleep quality (PSQI) (*r* = −0.16, *p* < 0.05). With regards to increasing pre-shift DBP the same relationships were found, with decreasing fatigue severity (*r* = −0.26, *p* < 0.01) and better overall sleep quality (*r* = −0.17, *p* < 0.05). Finally, post-shift DBP was negatively correlated with fatigue severity (*r* = −0.26, *p* < 0.01), overall sleep quality (*r* = −0.23, *p* < 0.01) and improved sleepiness risk (*r* = −0.16, *p* < 0.05). A subsequent stepwise regression analysis was found to be significant with the three variables (fatigue severity, overall sleep quality and sleepiness risk) together explaining 19% of the variance in post-shift DBP (F = 5.33, df = (6, 109), *p* < 0.01, *R* = 0.49, R^2^ = 0.24, adjusted R^2^ = 0.19).

**Table 1:** Shift work police officers’ (*n* = 206) demographics, questionnaires scores and cardiac measures.

Variable	Total (*n* = 206)		Men (*n* = 140)		Women (*n* = 66)	
Age (years)	31.63 ± 8.51		32.56 ± 8.68		29.64 ± 7.83 **^†^**	
Years of Service	6.44 ± 7.51		7.16 ± 8.14		4.92 ± 5.72 **^†^**	
WHR	−		0.89 ± 0.06		0.80 ± 0.06 **^†^**	
LAQ Part 1	13.27 ± 6.04		13.28 ± 6.35		13.26 ± 5.37	
PSQI	6.83 ± 3.58		7.02 ± 3.70		6.41 ± 3.29	
ESS	6.91 ± 4.12		6.99 ± 4.31		6.73 ± 3.71	
FSS	36.02 ± 10.36		34.74 ± 10.06		38.17 ± 10.62	
Cardiac Measures	*Pre Shift*	*Post Shift*	*Pre Shift*	*Post Shift*	*Pre Shift*	*Post Shift*
SBP (mmHg)	126.86 ± 12.33	128.11 ± 12.13 *	131.27 ± 10.40	131.66 ± 11.01	117.52 ± 10.87 **^†^**	120.59 ± 10.99 *^,**†**^
DBP (mmHg)	77.25 ± 8.92	76.96 ± 9.46	78.31 ± 8.81	78.08 ± 9.75	75.00 ± 8.79 **^†^**	74.58 ± 8.42 **^†^**

* Significantly different from pre to post-shift assessment (*p* < 0.05); **^†^** = Significantly different between the sexes (*p* < 0.05); **Key:** DBP = Diastolic Blood Pressure; ESS = Epworth Sleepiness Scale; FSS = Fatigue Severity Scale; LAQ = Lifestyle Appraisal Questionnaire; mmHg = millimetres mercury; PSQI = Pittsburgh Sleep Quality Index; SBP = Systolic Blood Pressure; WH*R* = Waist-Hip Ratio.

**Table 2:** Significant partial correlations between cardiac measures and other variables in corrected model **^+^**.

	Variables	*r*
Pre SBP	Fatigue Severity (FSS)	−0.26 **
Post SBP	Overall Sleep Quality (PSQI)	−0.16 *
	Fatigue Severity (FSS)	−0.25 **
Pre DBP	Overall Sleep Quality (PSQI)	−0.17 *
	Fatigue Severity (FSS)	−0.26 **
Post DBP	Overall Sleep Quality (PSQI)	−0.23 **
	Fatigue Severity (FSS)	−0.26 **
	Sleepiness Risk (ESS)	−0.16 *

**^+^** = accounting for age, waist-hip ratio and lifestyle risk factors, * = *p* < 0.05, ** *p* < 0.01; **Key:** DBP = Diastolic Blood Pressure; ESS = Epworth Sleepiness Scale; FSS = Fatigue Severity Scale; PSQI = Pittsburgh Sleep Quality Index; SBP = Systolic Blood Pressure.

### 3.3. Occupational Rank Comparisons

MANCOVA, with LSD Post-Hoc analysis, were performed to identify differences between police officers based on their occupational rank (Table 3), which included probationary constables (*n* = 42), constables (*n* = 86), senior constables (*n* = 42) and sergeants (*n* = 36) in an increasing hierarchy. As detailed in Section 2.4, the effects of age, WHR and lifestyle risk factors (LAQ) were accounted for in the analyses. Mean years of active service in the Police Force was found to significantly differ between the ranks (F_(4,201)_ = 107.33, *p* < 0.001), with sergeants serving in the NSW Police Force for an average 19.10 ± 7.25 years which was significantly longer than the 0.51 ± 0.04 years served by probationary constables (*p* < 0.001), constables’ 2.48 ± 1.15 years (*p* < 0.001) and senior constables’ 9.60 ± 3.84 years (*p* < 0.001). Again, senior constables’ mean years of service was also significantly longer than probationary constables (*p* < 0.001) and constables (*p* < 0.001), with the latter two also significantly different in mean years of service (*p* < 0.05).

With respect to BP measures taken before and after shift, a number of differences were identified between the police officers based on occupational rank. Although the MANCOVA for pre-shift SBP (F_(4,201)_ = 4.12, *p* < 0.01), pre-shift DBP (F_(4,201)_ = 12.64, *p* < 0.001) and post-shift DBP (F_(4,201)_ = 8.83, *p* < 0.001) were found to be significant, there were no elucidated differences between the ranks after Post-Hoc analysis. Similarly, post-shift SBP (F_(4,201)_ = 6.19, *p* < 0.001) was also found to significantly differ between the ranks, with probationary constables’ average of 128.83 ± 10.70 mmHg identified as significantly higher than constables’ 127.02 ± 11.58 mmHg (*p* < 0.05) and senior constables’ 125.62 ± 12.39 mmHg (*p* < 0.05).

**Table 3:** Significant differences between police officers based on occupational rank in corrected model (MANCOVA) **^+^**.

Source of Variance		*df*	*F*
*Occupational Rank:*	*Years of Service*	4	107.33 **
Probationary Constable (*n* = 42)	Pre SBP	4	4.12 *
Constable (*n* = 86)	Post SBP	4	6.19 **
Senior Constable (*n* = 42)	Pre DBP	4	12.64 **
Sergeant (*n* = 36)	Post DBP	4	8.83 **
	Error	201	

**^+^** = accounting for age, waist-hip ratio and lifestyle risk factors, * *p* < 0.01, ** = *p* < 0.001; **Key:** DBP = Diastolic Blood Pressure; SBP = Systolic Blood Pressure.

## 4. Discussion

### 4.1. Shift Work and Blood Pressure

This study of general duties police officers working twelve hour shift work sought to determine changes in their BP and associations with overall sleep quality and fatigue, as well as identify whether differences exist in these variables based on sex, shift and occupational rank. While there was a significant increase in SBP after shift work, there was no change in DBP. These findings conform to available literature that shift work may have the propensity to impede BP regulation and increase the risk of hypertension, potentially through circadian rhythm dysregulation [6,8]. Continuing activity across a period of habitual rest, which night shift work requires, will disrupt the body’s natural diurnal cycle and impact cardiometabolic health [11], thereby increasing BP [7]. Alternatively, the social and chronobiological burden of shift work may manifest as sleep disturbances and result in poor sleep quality and fatigue [9], observed to predominate within this sample. The cardiovascular impacts of impaired sleep has been observed in acute sleep deprivation studies [24], wherein individuals experienced increased sympathetic and decreased parasympathetic cardiac modulation, although no direct changes in BP were recorded.

While there is well documented literature for emergency responders’ increased risk of hypertension [25], the results of this study are more likely attributable to the negative cardiovascular effects of shift work. The reason for this is that if the occupation of policing was responsible for the observed changes in BP, both male and female subjects should have been affected, while only the female police officers were found to experience a significant increase in SBP after their shift. However, the practical clinical significance of this finding may be considered negligible as determined by the effect size. Despite this, these observations are similar to another study reporting that female nurses who had worked over 6 years were negatively affected by the circadian disrupting effects of shift work [26]. Although the females assessed in the present study had an average of 5 years’ service in the NSW Police Force, this may be evidence that shift work is directly affecting their BP and requires further research to determine to what extent this could be affecting their cardiovascular health.

A number of significant associations with BP were found by this study. To account for the established effects of lifestyle risk factors, central adiposity (WHR) and age upon cardiovascular health [22,23,27,28] these variables were treated as covariates in the statistical analyses (Section 2.4). Despite accounting for this, novel relationships were identified between increasing SBP and DBP, both pre and post-shift, with decreasing fatigue severity (FSS) and better sleep quality (PSQI). This conflicts with available literature as usually those with poor sleep quality have lesser-dips in BP and hence have a higher prevalence of hypertension [29]. However, those with chronic fatigue syndrome have been found to have impaired BP variability [14], leading to reduced overall BP. Further, those with less interrupted sleep (also assessed as a component of the PSQI, thereby contributing to the global score) will experience more dips in blood pressure which is beneficial for cardiovascular health [13]. Alternatively, female police officers were also found to have worse overall FSS scores; as their SBP was significantly lower compared to males, this could also be responsible for the relationship found indicating further research is required to confirm these preliminary findings.

Although police officers’ questionnaire scores were similar across the cohort, a number of significant differences were identified based on sex, shift and occupational rank. The higher BP values observed in males compared to females were to be expected, due to established differences in musculoskeletal mass and hormone levels between the sexes [30]. However, the significant difference in SBP between the day and night shift officers suggests it may be a potential confounder, due to the circadian rhythmicity of BP, and as such must be addressed in future research (Section 4.3). By comparison, despite accounting for the age-related changes in BP, due to stiffening of the arterial tree and increasing vascular resistance [27,28], there were significant differences in cardiovascular measures between the ranks. Additional information such as on-shift responsibilities may have accounted for these differences and should be recorded and considered in future studies. However, literature suggests shift work is an independent risk factor of hypertension [12], so coupled with the significant increase in females’ SBP, future research is required to identify to what extent current roster systems affect cardiovascular health in general duties police officers.

### 4.2. Shift Work, Sleep and Fatigue

Within this police officer sample, sleepiness levels (ESS) were within normal ranges, while poor sleep quality (PSQI) predominated with fatigue having a strong impact (FSS). Despite often being used synonymously, due to their symptomatic similarities, sleepiness and fatigue have been found to be distinct [4], hence their separate presentation in this cohort. These findings conform to available literature, that shift work will have a negative impact on sleep quality and quantity, thereby potentiating fatigue [9]. Although the NSW Police Force already employs a rapid, clockwise rotating shift system [2], both positive traits per available literature [31], poor sleep health and fatigue remain concerns for the occupation of policing in developed nations [32].

### 4.3. Limitations

Limitations with respect to this project include its cross-sectional design, which may have limited the scope of the findings. Secondly, the time of assessment (day or night shift) may have influenced subject’s BP measurements, and as such must be assessed on both a day and night shift in future. Ideally the incorporation of a 24 h ambulatory monitor would account for the dynamic nature of BP [16], and allow a more detailed insight into the suspected cardiovascular effects of shift work. Finally, additional information such as activities during shift and position within the roster cycle (first or second day/night shift) must be recorded in future, as this may have influenced the results.

## 5. Conclusions

In conclusion, shift work has been identified to have an increased prevalence of various pathologies which may be further exacerbated by the inherently demanding occupation of policing. However, heavy contention exists as to whether shift work has a direct effect upon BP regulation. This study has found that female police officers experience a significant increase in SBP after shift work, while poor sleep quality and fatigue appear to predominate within the police sample. With further research, the potential benefits of this study include lifestyle and fatigue management strategies, such as sleep health education or napping strategies. Suggestions such as these may ameliorate some of the concerns identified by this research, which would improve the occupational health and safety of the NSW Police Force, as well as ensure the ongoing safety needs of the wider community.
